# Reexploring the STRESS Trial: Subgroup Postoperative Outcomes Following Methylprednisolone for Infant Heart Surgery

**DOI:** 10.1007/s00246-025-03875-9

**Published:** 2025-05-02

**Authors:** Sudeep D. Sunthankar, Kevin D. Hill, Jeffrey P. Jacobs, H. Scott Baldwin, Marshall L. Jacobs, Jennifer S. Li, Eric M. Graham, Ashraf M. Resheidat, Venugopal Amula, Mark S. Bleiweis, Eric L. Wald, Pirooz Eghtesady, John P. Scott, Brett R. Anderson, Michael F. Swartz, Alexis Benscoter, William Ravekes, Prince J. Kannankeril

**Affiliations:** 1 Division of Pediatric Cardiology and Center for Pediatric Precision Medicine, Department of Pediatrics, Vanderbilt University Medical Center, 2220 Children’s Way; Suite 5230, Nashville, TN 37232 USA; 2 Division of Pediatric Cardiology, Duke University Medical Center, Durham, NC USA; 3 Division of Cardiac Surgery, University of Florida, Gainesville, FL USA; 4 Division of Cardiac Surgery, Johns Hopkins School of Medicine, Baltimore, MD USA; 5 Division of Pediatric Cardiology, Medical University of South Carolina, Charleston, SC USA; 6 Division of Pediatric Cardiac Anesthesiology, Texas Children’s Hospital, Baylor College of Medicine, Houston, TX USA; 7 Division of Critical Care, Department of Pediatrics, Primary Children’s Hospital, Salt Lake City, UT USA; 8 Division of Pediatric Cardiology, Northwestern University Feinberg School of Medicine, Chicago, IL USA; 9 Division of Cardiac Surgery, Washington University School of Medicine, St. Louis, MO USA; 10 Division of Pediatric Cardiology, Medical College of Wisconsin, Madison, WI USA; 11 Division of Pediatric Cardiology, Icahn School of Medicine at Mt Sinai, New York, NY USA; 12 Division of Cardiac Surgery, University of Rochester, Rochester, NY USA; 13 Division of Pediatric Cardiology, Cincinnati Children’s Hospital, Cincinnati, OH USA; 14 Division of Pediatric Cardiology, Johns Hopkins School of Medicine, Baltimore, MD USA

**Keywords:** Congenital heart disease, Cardiac surgery, Critical care, Methylprednisolone

## Abstract

Objective Assess the association between intraoperative methylprednisolone and specific postoperative outcomes among subgroups undergoing infant heart surgery. Design: Subpopulation analyses of The Steroids to Reduce Systemic Inflammation after Infant Heart Surgery trial, a double-blind randomized placebo-controlled trial. Setting: 24 congenital heart centers. Patients: Infants (< 1 year old) undergoing heart surgery with cardiopulmonary bypass. Patients stratified by Society of Thoracic Surgeons–European Association for Cardio-Thoracic Surgery Congenital Heart Surgery (STAT) Mortality Category, age, gestational age, and presence of chromosomal or syndromic diagnosis (CSD). Interventions: Methylprednisolone (30 mg/kg) versus placebo administered into cardiopulmonary bypass pump-priming fluid. Measurements and Main Results: Outcomes included death, heart transplantation, mechanical circulatory support, reinterventions, and hospital length of stay. Ranked composite outcome (death, transplant, or one of 13 major complications) was compared between placebo and methylprednisolone for each subgroup using the win ratio. Methylprednisolone did not reduce odds of death, transplant, or mechanical circulatory support for any subgroup. Those receiving methylprednisolone had fewer catheterization or surgical reinterventions after STAT Category 1–3 operations [OR 0.50 (0.29–0.86)]; and fewer reoperations for bleeding among patients undergoing STAT Category 1-3 operations [OR 0.28 (0.09–0.87)], term infants [OR 0.30 (0.12–0.76)], and those without CSD [OR 0.22 (0.07–0.68)]. Length of stay was no different between methylprednisolone versus placebo. Those without chromosomal or syndromic diagnosis demonstrated a favorable association for methylprednisolone [win ratio 1.28 (1.01–1.61)] for the composite outcome. Conclusion: Exploratory subpopulation analyses, although underpowered, suggest that methylprednisolone is not associated with significant harm and may benefit certain subpopulations

**Supplementary Information:**

The online version contains supplementary material available at 10.1007/s00246-025-03875-9.

## Introduction

Pediatric congenital heart disease (CHD) surgery incurs significant risk of morbidity and mortality, particularly during the postoperative hospitalization [1]. Previous work has investigated potential risk-mitigating interventions [2–10]. One area of interest has been the use of perioperative glucocorticoids to reduce post-cardiopulmonary bypass inflammation and subsequent postoperative sequelae [2–7]. The Steroids to Reduce Systemic Inflammation after Infant Heart Surgery (STRESS) trial randomized 1200 infants undergoing cardiac surgery with cardiopulmonary bypass to prophylactic intraoperative methylprednisolone versus placebo [2]. In these analyses, we aim to report the association of prophylactic intraoperative methylprednisolone with key postoperative outcomes in select subpopulations.

## Methods

### Parent Study

The STRESS trial was a multicenter, prospective, double-blind, randomized, placebo-controlled trial. The trial was performed within the existing Society of Thoracic Surgeons Congenital Heart Surgery (STS-CHSD) national registry with participation from 24 CHD institutions across the United States [2]. The protocol for the study was approved by the Vanderbilt University institutional review board (IRB# 170,859; approved: 06/20/2017) and at each participating site [11]. Written informed consent was obtained from a parent or legal guardian before enrollment. Each participating center performed block randomization to ensure equal allotment to intraoperative methylprednisolone versus placebo within each center.

### Subgroups and Outcomes

Participants were < 1 year of age and undergoing cardiac surgery with cardiopulmonary bypass. Of the 1263 patients randomized, 1200 received placebo or methylprednisolone as assigned and were included in these analyses. The eight subgroups in which placebo and methylprednisolone were compared were: Society of Thoracic Surgeons–European Association for Cardio-Thoracic Surgery Congenital Heart Surgery (STAT) Mortality Category [12] 1–3, STAT 4–5, neonate (0–30 days at time of operation), non-neonate (31–365 days old), premature infants (< 37 weeks at birth), term gestational age infants, those with a chromosomal or syndromic diagnosis (CSD), and those without CSD. CSD was defined by the 2016 STS data specifications. These groups were chosen as they have been considered risk factors for worse outcomes in patients undergoing CHD surgery [12–17]. Furthermore, these stratifying features are reflected in the preoperative status of the patient; therefore, if beneficial or harmful associations are present, based on results of this work, use of intraoperative methylprednisolone could be tailored accordingly. Individuals with corrected gestational age < 37 weeks at time of surgery were excluded from enrollment, thus, the prematurity dichotomization is based on gestational age at birth and not reflective of corrected gestational age at time of surgery. Missing data were handled by multiple imputations for stratifying variables (STAT Category *n = 3*, prematurity status *n = 3*, and CSD *n =* 1). The deidentified STRESS trial database, for secondary analyses, coded CSD as Down Syndrome, DiGeorge Syndrome, or not-specified. This latter category was created to protect patient anonymity in secondary analyses.

We sought to evaluate the associations of prophylactic intraoperative methylprednisolone with respect to outcomes in the aforementioned subgroups. Outcomes of interest within these analyses were defined in the STRESS trial study protocol and included (1) death, transplant, or mechanical circulatory support (D/Tx), (2) unplanned cardiac reinterventions, (3) reoperation for bleeding, (4) postoperative length of stay (PLOS), and (5) the overall ranked composite outcome from the parent study.

D/Tx included inpatient mortality, 30-day mortality (inpatient or outpatient), heart transplantation during postoperative hospitalization, or requirement of postoperative mechanical circulatory support. Unplanned cardiac reinterventions included catheterization and surgical procedures, which occurred (1) within 30 days of primary surgical operation regardless of hospitalization status or (2) after 30 days but within the same hospitalization as the primary surgical operation. Procedures such as delayed sternal closure, ECMO decannulation, and removal of central line catheter were coded as planned interventions and thus were not included in this variable. Any postoperative reoperation required for bleeding was excluded from the catheterization or surgical reintervention outcome and recorded separately. To provide anonymity for the study participants within secondary trial analyses, continuous variables, such as PLOS and the ranked composite outcome, were reorganized into ordered categorical variables in a deidentified data set. PLOS was categorized into 5-day increments from 0 to 70 days, 10-day increments from 70 to 100 days, and > 100 days. The ranked composite outcome was comprised of 16 levels of priority (eTable 1 in Supplement 1) [2]. The ranked composite outcome assigns the highest rank based on the outcome(s) for each patient. If none of the individual major complications occurred, patients were ranked based on length of stay in ten day intervals (e.g., rank of 80 for PLOS of 81–90 days, rank of 70 for PLOS of 71–80 days). The effect of intraoperative methylprednisolone on outcomes of interest was investigated in the two specific syndromic diagnoses available (Down Syndrome and DiGeorge Syndrome).

### Statistical Methods

Pearson’s Χ^2^ was used to compare categorical variables. Covariate adjusted logistic regression was used to calculate odds ratio (OR) and 95% confidence intervals (95% CI) for binary outcomes of interest (D/Tx, bleeding reoperation, surgical/catheterization reintervention) with respect to methylprednisolone versus placebo. These analyses adjusted for STAT Mortality Category, cardiopulmonary bypass time, and weight to minimize confounding from known operative risk factors. Ordinal regression was used to assess a difference in PLOS with methylprednisolone versus placebo. PLOS was censored for death and heart transplantation in this analysis to not misrepresent early mortality or transplantation as PLOS. The association between the ranked composite end point score and intraoperative methylprednisolone was measured by the win ratio [18]. The win ratio allows for reporting treatment effect in a ranked composite outcome accounting for differing levels of clinical severity. The unmatched win ratio is calculated by creating all possible pairs comprised of one patient from the treatment group and one patient from the placebo group and dividing the number of pairs in which the patient from the treatment group had a better outcome by the number of pairs in which the patient from the placebo group had a better outcome [19]. A two-tailed *P*-value < 0.05 was considered significant. Given the exploratory nature of the work, correction for multiple comparisons was not performed; however, the authors provided adjusted odds ratio for outcomes of interest to provide transparency for tested associations. Statistical analysis and figure creation was completed with Stata version 16 (Stata Corporation, College Station, Texas, USA).

## Results

### Study Population

The 1200 patients who received placebo or prophylactic intraoperative methylprednisolone as assigned by randomization were included in the analysis (eFigure 1 in Supplement 1). In the entire cohort, 46% were female. Neonates were more likely to undergo higher mortality risk (STAT 4–5) operations than non-neonates. Among all ages, those born prematurely were more likely to undergo lower mortality risk (STAT 1–3) operations than term gestational infants. CSD was more common in non-neonates and those of premature gestational age. Balance between the treatment and placebo arms was maintained for each subgroup. Demographic features are shown in Table 1.

### Outcomes

**Fig. 1:**
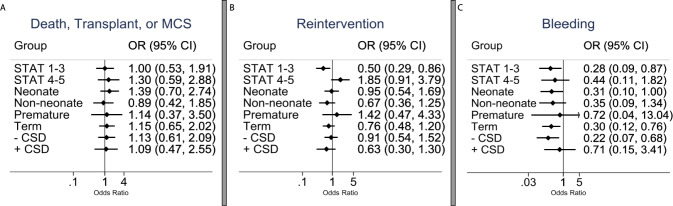
Adjusted odds ratio (95% confidence interval) for binary outcomes of interest: (A) death, heart transplantation, and mechanical circulatory support, (B) Reintervention with heart catheterization and/or cardiac surgery, but excludes bleeding reoperations, and (C) Reoperaition due to bleeding. Odds ratio adjusted for STAT mortaltiy category, operative weight, and cardiopulmonary bypass time. *Mechanical Circulatory Support (MCS)*

D/Tx occurred in 6% (76/1200) of the entire cohort. Prophylactic intraoperative methylprednisolone did not provide a benefit for death, transplant, or need for mechanical circulatory support in any of the eight subgroups (eTable 2–5 in Supplement 1; Fig. 1).

In the overall cohort, 2% (28/1200) of patients required a surgical reoperation for bleeding. Apart from reoperations for bleeding, unplanned catheterization or surgical reinterventions occurred in 9% (104/1200) for the entire cohort. When stratified by STAT Category, patients undergoing STAT Mortality Category 1–3 operations had lower odds of requiring a reoperation for bleeding [OR 0.28 (0.09–0.87), *P* = 0.028] or an unplanned cardiac reintervention [OR 0.50 (0.29–0.86), *P* = 0.012] if they received methylprednisolone rather than placebo. No other subgroup demonstrated a reduction in unplanned cardiac reinterventions when comparing methylprednisolone to placebo. Those with term birth gestational age [OR 0.30 (0.12–0.76), *P* = 0.012], and those without CSD [OR 0.22 (0.07–0.68), *P* = 0.009] all had reduced odds of requiring a reoperation for bleeding following cardiac surgery with methylprednisolone versus placebo (eTable 2–5 in Supplement 1; Fig. 1).

### Length of Stay and Ranked Composite Outcome

**Fig. 2:**
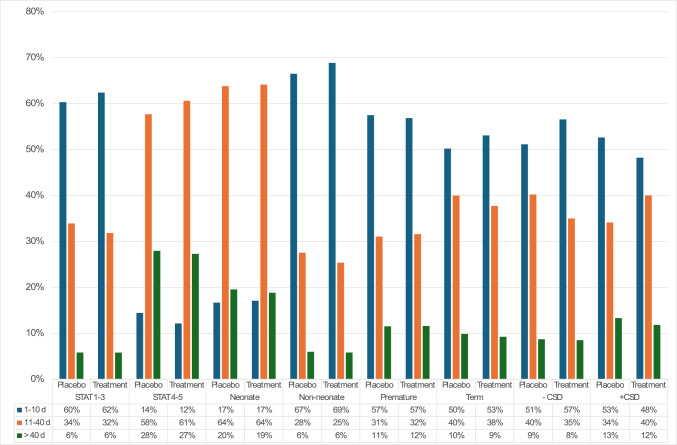
Postoperative length of stay (PLOS) censored for death or transplant. No difference for PLOS between placebo and methylprednisolone by ordinal regression for any subgroup. PLOS bins were condensed for graphic visualization. Full data available in eTable 6 in Supplement 1

**Fig. 3:**
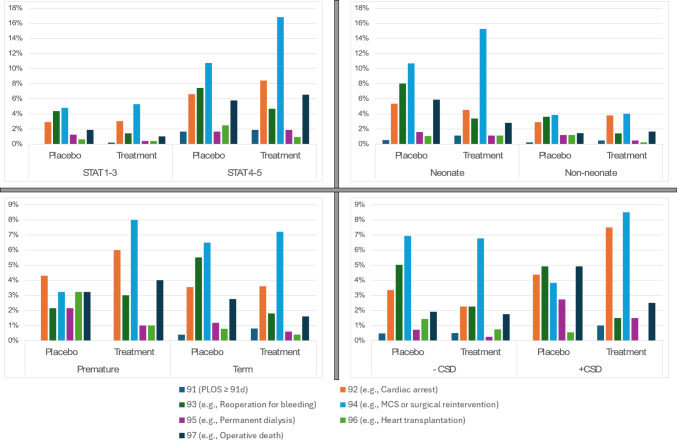
Distribution of ranked composite outcome for those with a score ≥ 91 categorized by treatment versus placebo for each subgroup

**Fig. 4:**
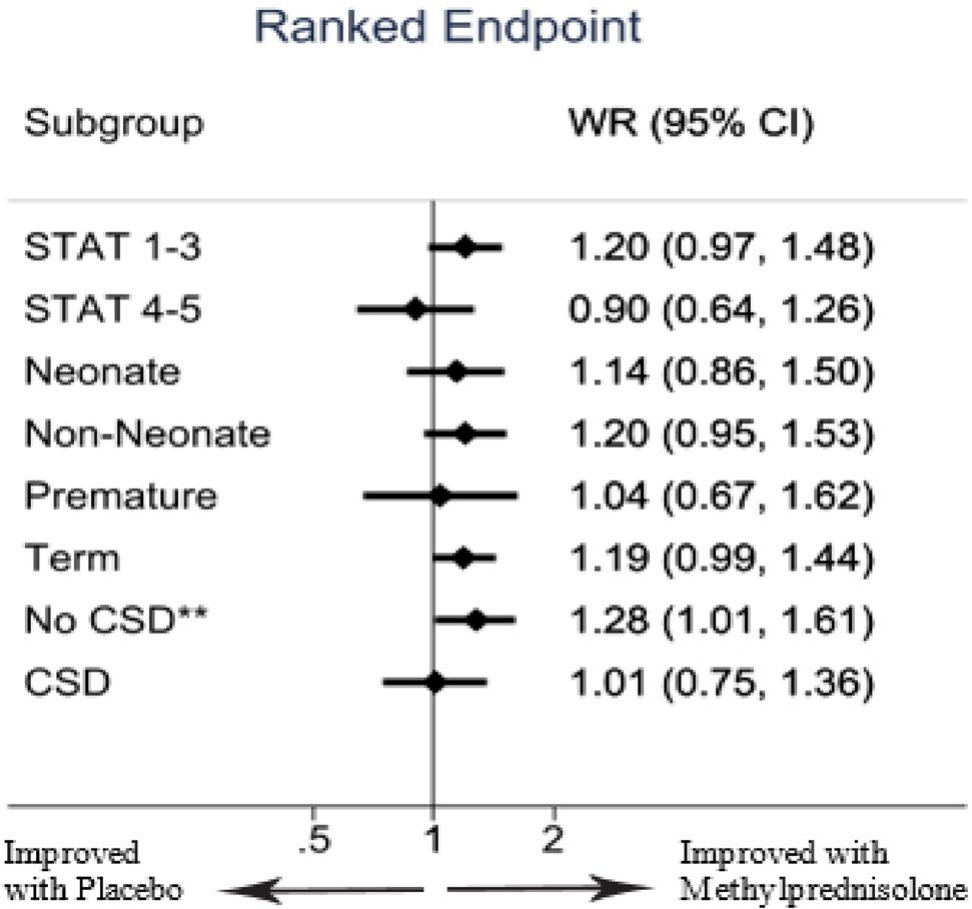
Unmatched win ratio (WR) and 95% CI for the ranked composite outcome for each subgroup. WR > 1 predicts benefit of methylprednisolone. **P < 0.05. Full description of ranked outcomes are included in eTable 1 in Supplement 1. Ranked outcomes were based on priority assigned by parent manuscript [2]

For the overall cohort, PLOS between the methylprednisolone and placebo arms was no different. There was no difference in PLOS between methylprednisolone and placebo for any of the eight subgroups (Fig. 2, eTable 6 in Supplement 1). Distributions for ranked composite outcome scores ≥ 91 are shown in Fig. 3. The win ratio demonstrated a benefit with intraoperative methylprednisolone for those without CSD (win ratio 1.28 [1.01–1.61]; Fig. 4). Point estimates favored methylprednisolone over placebo in other perceived lower risk subpopulations (STAT 1–3: WR 1.20 [0.97 – 1.48], non-neonates: WR 1.2 [0.95–1.53], and term gestational age infants: WR 1.19 [0.99–1.44]).

### Down Syndrome and DiGeorge Syndrome

Among the 383 patients with CSD, 202 had a diagnosis of Down Syndrome and 47 had a diagnosis of DiGeorge Syndrome. D/Tx were rare in both subgroups (eTable 7 in Supplement 1). There was no difference in surgical or catheterization reinterventions with intraoperative steroids for either diagnosis group. Among patients with Down Syndrome there were similar rates of PLOS > 30 days with placebo versus methylprednisolone. Among patients with DiGeorge Syndrome, 38% (11/29) had PLOS > 30 days with placebo compared to 22% (4/18) treated with intraoperative methylprednisolone; however, this is in the setting of a small subset population. Win ratio did not indicate a benefit from intraoperative methylprednisolone for either diagnostic group with respect to the composite outcome [Down Syndrome: 0.91 (0.58–1.45); DiGeorge Syndrome: 1.04 (0.47–2.29)].

## Discussion

In this subgroup analysis of the STRESS trial, infants with prophylactic intraoperative methylprednisolone had fewer reoperations for bleeding within several subgroups of interest: STAT Mortality Categories 1–3, term gestational age, and those without CSD. Unplanned catheterization or surgical reinterventions were less frequent in those undergoing STAT Mortality Category 1–3 operations when receiving methylprednisolone compared to placebo. Those without CSD had an improved win ratio, with respect to the ranked composite end point, when receiving intraoperative methylprednisolone compared to placebo. Intraoperative treatment with methylprednisolone showed no benefit, compared to placebo, with respect to either death, transplant, or mechanical circulatory support or PLOS in any of the eight subgroups.

The results presented here align with those of previous randomized controlled trials in infants undergoing cardiac surgery. The Dexamethasone in Pediatric Cardiac Surgery (DECISION) trial randomized 394 infants (< 12 months old) to intraoperative dexamethasone versus placebo [5]. Graham *et. al* included 176 neonates (≤ 31 days old) randomized to intraoperative methylprednisolone versus placebo [3]. Both of these works demonstrated no difference in mortality or need for ECMO, with prophylactic steroid administration. Similar to our study (mortality 1%; MCS 5%), occurrences of mortality and MCS in the referenced articles were rare and limit the ability to detect a meaningful treatment effect. Larger randomized controlled trials in adult populations testing the benefit of glucocorticoids for patients undergoing cardiac surgery with cardiopulmonary bypass have demonstrated no mortality benefit [20, 21].

The occurrence of residual lesions following primary cardiac surgery is multifactorial, being variously associated with operative complexity, anatomical considerations, preoperative patient status, and adequacy of repair [22–24]. Previous prospective work studying common CHD surgeries demonstrated that residual surgical lesions requiring surgical reintervention portend worse postoperative outcomes [22]. The stratified subgroup analysis by STAT Mortality Category demonstrated a lower odds of unplanned catheterization or surgical reintervention for the lower mortality risk (STAT Mortality Category 1–3) operations with no benefit in the higher mortality risk (STAT Mortality Category 4–5) operations among patients randomized to methylprednisolone rather than placebo. The authors hypothesize that the discrepancy in benefit between the two stratifications reflects the reduced contribution from anatomical and operative complexity in STAT Mortality Category 1–3 operations. Reinterventions are often pursued during postoperative hospitalization in patients who have hemodynamic instability necessitating the care team reintervene to mitigate any possible contribution from residual anatomical lesions. Thus, the authors propose prophylactic intraoperative steroids, by reducing the overall inflammatory response to cardiopulmonary bypass, may contribute to hemodynamic stability, particularly during the early postoperative course. The magnitude of the impact of a residual defect may be less, and better tolerated, if overall hemodynamics are more robust, and thus fewer reinterventions may be necessitated. Intraoperative steroids may also reduce postoperative cardiac inflammation and provide a more robust cardiac index in the postoperative interval allowing for improved healing and less frequent breakdown of surgically repaired sites [25, 26]. Previous reports have described a reduced hemoglobin affinity for oxygen following methylprednisolone administration; therefore, allowing for reduced tissue hypoxia [27, 28]. Further translational investigation into the metabolic and cellular-level benefits of glucocorticoids may further elucidate the advantages of methylprednisolone in the perioperative space.

The authors acknowledge that while there was a reduction in reoperations for bleeding in the overall cohort and among several subgroups, this is in the context of a low event rate. A meta-analysis including studies of adults undergoing cardiac surgery with cardiopulmonary bypass randomized to placebo or steroid demonstrated a modest, but statistically significant, reduction in postoperative bleeding; however, reoperations for bleeding were not reported [29]. Cardiopulmonary bypass is known to cause dysregulation of the coagulation cascade and subsequent bleeding complications post cardiac surgery [30]. Reports have demonstrated that glucocorticoids increase clotting factor levels and fibrinogen compared to placebo in healthy controls [31]. Increases in these factors may mitigate bleeding and need for reoperation. Of note, the study protocol [2, 11] did not dictate intraoperative or postoperative blood product administration and this was at the discretion of the provider and/or institution. This could influence rates of bleeding reoperations; individual center rates of reoperations were not available in this analysis.

PLOS, censored for D/Tx, was similar between those receiving intraoperative methylprednisolone versus placebo within each subgroup. Graham et al*.* also showed no difference in intensive care unit or total PLOS with methylprednisolone versus placebo [3]. The authors acknowledge that PLOS following infant cardiac surgery is multifactorial and a singular medication is unlikely to alleviate the many possible contributing features. However, neutral findings are also important given the benefit in other areas of interest. The win ratio, analyzing the ranked composite outcome, demonstrated a benefit of intraoperative methylprednisolone compared to placebo for those without CSD. The other perceived lower risk groups (STAT Mortality Category 1–3, non-neonates, and term gestational age) had win ratios > 1 with lower range of 95% confidence interval approaching 1. Overall, these data are consistent with the win ratio presented in the parent manuscript for methylprednisolone in the overall cohort [1.15 (1.00–1.32)] [2]. It is encouraging that no subgroup had a notably worse outcome with methylprednisolone; suggesting that there is a neutral to beneficial overall effect of intraoperative methylprednisolone within these subpopulations.

Limitations of this study are inherently similar to those outlined in the original STRESS trial manuscript, including those inherent in the use of registry data [2]. Differences in institutional practice patterns must be considered in the interpretation of these data. Variations in threshold for reinterventions between institutions could affect outcomes of interest within this study. Intra- and postoperative protocols aimed at mitigating postoperative bleeding were at the discretion of the individual institution. Conceivably, these could affect reoperations for bleeding. Down Syndrome and DiGeorge Syndrome were the most common CSD in this cohort. No appreciable benefit was observed with prophylactic methylprednisolone in patients with either diagnosis. Excluding the aforementioned pair, classification of specific diagnoses was unavailable for review; therefore, we have only a partial understanding of how the severity of the CSD may have impacted the outcome profile of prophylactic intraoperative methylprednisolone. To protect patient anonymity in the deidentified dataset that is made available for secondary analyses, continuous variables, such as PLOS and ranked composite outcome score, were reorganized into ordered categorical variables. This may have come at the cost of variable granularity. Some outcomes of interest included in this analysis had low rates of occurrence, which should be factored into the interpretation of these findings. Large datasets are also capable of producing small confidence intervals, which infer small clinical differences as statistically significant. Secondary analyses of randomized controlled trials may be underpowered to detect true effects. The authors acknowledge this work is exploratory to investigate associated benefits and harms of methylprednisolone among specific subgroups. Thus, we encourage the reader to evaluate the statistical, but also clinical implications of these data.

## Conclusion

This subgroup analysis of the STRESS randomized placebo-controlled trial, which enrolled infants undergoing cardiac surgery, demonstrated that prophylactic intraoperative methylprednisolone, compared to placebo, had no increase in death or heart transplantation, mechanical circulatory support, or postoperative hospital length of stay. The methylprednisolone arm had fewer reoperations for bleeding in several subgroups; however, such events were rare overall. Win ratio suggested a benefit with methylprednisolone in those without CSD. Subpopulation analyses, although underpowered, demonstrate that prophylactic methylprednisolone is not associated with significant harm and may offer benefit for certain subgroups.

## Supplementary Information

Below is the link to the electronic supplementary material.

Supplementary file1 (DOCX 356 KB)

## Data Availability

No datasets were generated or analysed during the current study.

## Abbreviations

CHD: Congenital heart disease
CSD: Chromosomal or syndromic diagnosis
D/Tx: Death or heart transplantation
ECMO: Extracorporeal membrane oxygenation
MCS: Mechanical circulatory support
PLOS: Postoperative hospital length of stay
STAT: Society of thoracic surgeons–European association for cardio-thoracic surgery congenital heart surgery
STRESS: Steroids to reduce systemic inflammation after infant heart surgery
STS: Society of thoracic surgeons
VAD: Ventricular assist device

## References

[1] Jacobs JP, Mayer JE Jr, Pasquali SK, Hill KD, Overman DM, St Louis JD, Kumar SR, Backer CL, Tweddell JS, Dearani JA et al (2019) The society of thoracic surgeons congenital heart surgery database: 2019 update on outcomes and quality. Ann Thorac Surg 107(3):691–704 30641069 10.1016/j.athoracsur.2018.12.016

[2] Hill KD, Kannankeril PJ, Jacobs JP, Baldwin HS, Jacobs ML, O’Brien SM, Bichel DP, Graham EM, Blasiole B, Resheidat A et al (2022) Methylprednisolone for heart surgery in infants - a randomized. Controlled Trial N Engl J Med 387(23):2138–2149 36342116 10.1056/NEJMoa2212667 PMC9843240

[3] Graham EM, Martin RH, Buckley JR, Zyblewski SC, Kavarana MN, Bradley SM, Alsoufi B, Mahle WT, Hassid M, Atz AM (2019) Corticosteroid therapy in neonates undergoing cardiopulmonary bypass: randomized controlled trial. J Am Coll Cardiol 74(5):659–668 31370958 10.1016/j.jacc.2019.05.060 PMC6684326

[4] Graham EM, Atz AM, Butts RJ, Baker NL, Zyblewski SC, Deardorff RL, DeSantis SM, Reeves ST, Bradley SM, Spinale FG (2011) Standardized preoperative corticosteroid treatment in neonates undergoing cardiac surgery: results from a randomized trial. J Thorac Cardiovasc Surg 142(6):1523–1529 21600592 10.1016/j.jtcvs.2011.04.019 PMC3161127

[5] Lomivorotov V, Kornilov I, Boboshko V, Shmyrev V, Bondarenko I, Soynov I, Voytov A, Polyanskih S, Strunin O, Bogachev-Prokophiev A et al (2020) Effect of intraoperative dexamethasone on major complications and mortality among infants undergoing cardiac surgery: the decision randomized clinical trial. JAMA 323(24):2485–2492 32573670 10.1001/jama.2020.8133 PMC7312411

[6] Robert SM, Borasino S, Dabal RJ, Cleveland DC, Hock KM, Alten JA (2015) Postoperative hydrocortisone infusion reduces the prevalence of low cardiac output syndrome after neonatal cardiopulmonary bypass. Pediatr Crit Care Med 16(7):629–636 25901540 10.1097/PCC.0000000000000426

[7] Suominen PK, Keski-Nisula J, Ojala T, Rautiainen P, Jahnukainen T, Hastbacka J, Neuvonen PJ, Pitkanen O, Niemela J, Kaskinen A et al (2017) Stress-dose corticosteroid versus placebo in neonatal cardiac operations: a randomized controlled trial. Ann Thorac Surg 104(4):1378–1385 28434547 10.1016/j.athoracsur.2017.01.111

[8] Pasquali SK, Hall M, Li JS, Peterson ED, Jaggers J, Lodge AJ, Marino BS, Goodman DM, Shah SS (2010) Corticosteroids and outcome in children undergoing congenital heart surgery: analysis of the pediatric health information systems database. Circulation 122(21):2123–2130 21060075 10.1161/CIRCULATIONAHA.110.948737 PMC3013053

[9] Gibbons KS, Schlapbach LJ, Horton SB, Long DA, Beca J, Erickson S, Festa M, d’Udekem Y, Alphonso N, Winlaw D et al (2021) Statistical analysis plan for the NITric oxide during cardiopulmonary bypass to improve Recovery in Infants with Congenital heart defects (NITRIC) trial. Crit Care Resusc 23(1):47–58 38046394 10.51893/2021.1.OA4 PMC10692519

[10] Hoffman TM, Wernovsky G, Atz AM, Bailey JM, Akbary A, Kocsis JF, Nelson DP, Chang AC, Kulik TJ, Spray TL et al (2002) Prophylactic intravenous use of milrinone after cardiac operation in pediatrics (PRIMACORP) study. Prophylactic intravenous use of milrinone after cardiac operation in pediatrics. Am Heart J 143(1):15–21 11773907 10.1067/mhj.2002.120305

[11] Hill KD, Baldwin HS, Bichel DP, Butts RJ, Chamberlain RC, Ellis AM, Graham EM, Hickerson J, Hornik CP, Jacobs JP et al (2020) Rationale and design of the STeroids to REduce Systemic inflammation after infant heart surgery (STRESS) trial. Am Heart J 220:192–202 31855716 10.1016/j.ahj.2019.11.016 PMC7008076

[12] Jacobs ML, Jacobs JP, Thibault D, Hill KD, Anderson BR, Eghtesady P, Karamlou T, Kumar SR, Mayer JE, Mery CM et al (2021) Updating an empirically based tool for analyzing congenital heart surgery mortality. World J Pediatr Congenit Heart Surg 12(2):246–281 33683997 10.1177/2150135121991528

[13] Dimopoulos K, Constantine A, Clift P, Condliffe R, Moledina S, Jansen K, Inuzuka R, Veldtman GR, Cua CL, Tay ELW et al (2023) Cardiovascular complications of down syndrome: scoping review and expert consensus. Circulation 147(5):425–441 36716257 10.1161/CIRCULATIONAHA.122.059706 PMC9977420

[14] Sarno LA, Walters HL 3rd, Bondarenko I, Thomas R, Kobayashi D (2020) Significant improvements in mortality after the fontan operation in children with down syndrome. Ann Thorac Surg 109(3):835–841 31525348 10.1016/j.athoracsur.2019.07.085

[15] Alsoufi B, McCracken C, Shashidharan S, Deshpande S, Kanter K, Kogon B (2017) The Impact of 22q11.2 deletion syndrome on surgical repair outcomes of conotruncal cardiac anomalies. Ann Thorac Surg 104(5):1597–1604 28669502 10.1016/j.athoracsur.2017.04.019

[16] Best KE, Tennant PWG, Rankin J (2017) Survival, by birth weight and gestational age, in individuals with congenital heart disease: a population-based study. J Am Heart Assoc. 10.1161/JAHA.116.005213 28733436 10.1161/JAHA.116.005213 PMC5586271

[17] De Silvestro A, Reich B, Bless S, Sieker J, Hollander W, de Bijl-Marcus K, Hagmann C, Nijman J, Knirsch W (2024) European Association Brain in Congenital Heart D: Morbidity and mortality in premature or low birth weight patients with congenital heart disease in three European pediatric heart centers between 2016 and 2020. Front Pediatr 12:1323430 38665378 10.3389/fped.2024.1323430 PMC11043489

[18] Pocock SJ, Ariti CA, Collier TJ, Wang D (2012) The win ratio: a new approach to the analysis of composite endpoints in clinical trials based on clinical priorities. Eur Heart J 33(2):176–182 21900289 10.1093/eurheartj/ehr352

[19] Redfors B, Gregson J, Crowley A, McAndrew T, Ben-Yehuda O, Stone GW, Pocock SJ (2020) The win ratio approach for composite endpoints: practical guidance based on previous experience. Eur Heart J 41(46):4391–4399 32901285 10.1093/eurheartj/ehaa665

[20] Whitlock RP, Devereaux PJ, Teoh KH, Lamy A, Vincent J, Pogue J, Paparella D, Sessler DI, Karthikeyan G, Villar JC et al (2015) Methylprednisolone in patients undergoing cardiopulmonary bypass (SIRS): a randomised, double-blind, placebo-controlled trial. Lancet 386(10000):1243–1253 26460660 10.1016/S0140-6736(15)00273-1

[21] Dieleman JM, Nierich AP, Rosseel PM, van der Maaten JM, Hofland J, Diephuis JC, Schepp RM, Boer C, Moons KG, van Herwerden LA et al (2012) Intraoperative high-dose dexamethasone for cardiac surgery: a randomized controlled trial. JAMA 308(17):1761–1767 23117776 10.1001/jama.2012.14144

[22] Nathan M, Levine JC, Van Rompay MI, Lambert LM, Trachtenberg FL, Colan SD, Adachi I, Anderson BR, Bacha EA, Eckhauser A et al (2021) Impact of major residual lesions on outcomes after surgery for congenital heart disease. J Am Coll Cardiol 77(19):2382–2394 33985683 10.1016/j.jacc.2021.03.304 PMC8245007

[23] Sengupta A, Gauvreau K, Kohlsaat K, Colan SD, Newburger JW, Del Nido PJ, Nathan M (2022) Long-term outcomes of patients requiring unplanned repeated interventions after surgery for congenital heart disease. J Am Coll Cardiol 79(25):2489–2499 35738709 10.1016/j.jacc.2022.04.027

[24] Sengupta A, Gauvreau K, Kohlsaat K, Colan SD, Newburger JW, Del Nido PJ, Nathan M (2022) Intraoperative residual lesion score predicts predischarge major residual lesions and reinterventions following congenital heart surgery. J Am Coll Cardiol 80(12):1202–1204 36109112 10.1016/j.jacc.2022.07.009

[25] Whitlock RP, Young E, Noora J, Farrokhyar F, Blackall M, Teoh KH (2006) Pulse low dose steroids attenuate post-cardiopulmonary bypass SIRS. SIRS I J Surg Res 132(2):188–194 16566943 10.1016/j.jss.2006.02.013

[26] Niazi Z, Flodin P, Joyce L, Smith J, Mauer H, Lillehei RC (1979) Effects of glucocorticosteroids in patients undergoing coronary artery bypass surgery. Chest 76(3):262–268 380941 10.1378/chest.76.3.262

[27] Brada M, Robinson LA, Bellingham AJ (1980) The effect of methylprednisolone sodium succinate on erythrocyte and haemoglobin function. Clin Sci (Lond) 59(3):163–168 7000416 10.1042/cs0590163

[28] Bryan-Brown CW (1975) Tissue blood flow and oxygen transport in critically ill patients. Crit Care Med 3(3):103–108 1181095 10.1097/00003246-197505000-00005

[29] Whitlock RP, Chan S, Devereaux PJ, Sun J, Rubens FD, Thorlund K, Teoh KH (2008) Clinical benefit of steroid use in patients undergoing cardiopulmonary bypass: a meta-analysis of randomized trials. Eur Heart J 29(21):2592–2600 18664462 10.1093/eurheartj/ehn333

[30] Thiele RH, Raphael J (2014) A 2014 Update on coagulation management for cardiopulmonary bypass. Semin Cardiothorac Vasc Anesth 18(2):177–189 24876232 10.1177/1089253214534782

[31] Brotman DJ, Girod JP, Posch A, Jani JT, Patel JV, Gupta M, Lip GY, Reddy S, Kickler TS (2006) Effects of short-term glucocorticoids on hemostatic factors in healthy volunteers. Thromb Res 118(2):247–252 16005496 10.1016/j.thromres.2005.06.006

