# Supplementary material for: Reexploring the STRESS Trial: Subgroup Postoperative Outcomes Following Methylprednisolone for Infant Heart Surgery
Source: Pediatr Cardiol. 2025 May 2;47(3):1026–34. doi: 10.1007/s00246-025-03875-9 (PMC12354065; doi:10.1007/s00246-025-03875-9)
Supplement: Supplementary file 1 — Supplementary file1 (DOCX 356 KB) [file 246_2025_3875_MOESM1_ESM.docx]

**Supplementary Online Content**

**eTable 1**. Descriptions of Outcomes and Assignment of Rank

**eTable 2**. Outcomes for STAT Mortality Categories

**eTable 3**. Outcomes for Age Categories

**eTable 4**. Outcomes for Birth Gestational Age Categories

**eTable 5**. Outcomes for Chromosomal or Syndromic Diagnosis

**eTable 6**. Postoperative Hospital Length of Stay

**eTable 7**. Outcomes for Patients with Down Syndrome and DiGeorge Syndrome

**eFigure 1.** CONSORT Diagram

**eTable 1**. Descriptions of Outcomes and Assignment of Rank

| Rank | Description |
| --- | --- |
| 97 | Operative mortality (in-hospital mortality or mortality after hospital discharge but within 30 days of the exposure to study drug/placebo) |
| 96 | Heart transplant (during operative hospitalization) |
| 95 | Renal failure with permanent dialysis, neurologic deficit persistent at discharge, or respiratory failure |
| 94 | Post-operative mechanical circulatory support or unplanned surgical reintervention (not including reoperation for bleeding) |
| 93 | Reoperation for bleeding, unplanned delayed sternal closure, or post-op unplanned interventional cardiac catheterization |
| 92 | Post-op cardiac arrest, multi-system organ failure, renal failure  with temporary dialysis) , or prolonged ventilator support (> 7 days) |
| 91 | Post-operative length of stay > 90 days |
| 80 | Length of stay 81-90 days |
| 70 | Length of stay 71-80 days |
| 60 | Length of stay 61-70 days |
| 50 | Length of stay 51-60 days |
| 40 | Length of stay 41-50 days |
| 30 | Length of stay 31-40 days |
| 20 | Length of stay 21-30 days |
| 10 | Length of stay 11-20 days |
| 0 | Length of stay 1-10 days |

**eTable 2**. Outcomes for STAT Mortality Categories

|  | STAT Category 1-3 | | | STAT Category 4-5 | | |
| --- | --- | --- | --- | --- | --- | --- |
|  | Placebo (n=478) | Treatment (n=491) | Difference  [95% CI] | Placebo (n=121) | Treatment (n=107) | Difference  [95% CI] |
| 30-day mortality | 5 (1.0%) | 5 (1.0%) | 0.1 [-1.2 to 1.3]% | 1 (1%) | 4 (4%) | -2.9 [-6.7 to 1.0]% |
| Death or heart transplantation | 12 (2.9%) | 7 (1.4%) | 1.5 [-0.1 to 3.3]% | 10 (8.2%) | 8 (7.5%) | 0.8 [-6.3 to 7.9]% |
| Mechanical Circulatory Support | 13 (2.9%) | 15 (3.0%) | -0.1 [-0.2 to 0.2]% | 11 (9%) | 16 (15%) | -5.8 [-14.3 to 2.6]% |
| Catheterization or surgical reintervention | 41 (8.5%) | 23 (4.7%) | 3.9 [0.8 to 7.0]% | 16 (13.2%) | 24 (22.4%) | -9.2 [-19.1 to 0.7]% |
| Bleeding reoperation | 13 (2.7%) | 4 (0.8%) | 1.9 [0.1 to 3.5]% | 8 (6.6%) | 3 (2.8%) | 3.8 [-1.8 to 9.4]% |
| Ranked end point ≥ 91 | 76 (16.3%) | 58 (12.0%) | 4.3 [-0.1 to 8.6]% | 44 (36.4%) | 44 (41.1%) | -4.8 [-17.5 to 8.0]% |

eTable 2. Categorical variables presented as count (%). Abbreviations: STAT, Society of Thoracic Surgeons–European Association for Cardio-Thoracic Surgery Congenital Heart Surgery.

**eTable 3**. Outcomes for Age Categories

|  | Neonate | | | | Non-Neonate | | | |
| --- | --- | --- | --- | --- | --- | --- | --- | --- |
|  | Placebo (n=187) | Treatment (n=177) | Difference  [95% CI] | Placebo (n=414) | | Treatment (n=422) | Difference  [95% CI] |  |
| 30-day mortality | 4 (2.1%) | 2 (1.1%) | 1.0 [-1.6 to 3.6]% | 2 (0.5%) | | 7 (1.7%) | 1.1 [-2.6 to 0.2]% |  |
| Death or heart transplantation | 13 (6.9%) | 7 (4.0%) | 3.0 [-1.7 to 7.7]% | 11 (2.7%) | | 8 (1.9%) | 0.8 [-1.2 to 2.8]% |  |
| Mechanical Circulatory Support | 15 (8.1%) | 20 (11.3%) | -3.2 [-9.4 to 2.8]% | 10 (2.4%) | | 11 (2.6%) | -0.2 [-2.3 to 1.9]% |  |
| Catheterization or surgical reintervention | 31 (16.5%) | 28 (15.8%) | 0.8 [-6.8 to 8.3]% | 26 (6.2%) | | 19 (4.5%) | 1.8 [-1.3 to 4.8]% |  |
| Bleeding reoperation | 13 (7.0%) | 4 (2.2%) | 4.7 [0.4 to 9.0]% | 8 (2.0%) | | 3 (0.7%) | 1.2 [-0.3 to 2.8]% |  |
| Ranked end point ≥ 91 | 62 (33.2%) | 52 (29.4%) | 3.8 [-5.8 to 13.3]% | 60 (14.5%) | | 51 (12.1%) | 2.4 [-2.2 to 7.0]% |  |

eTable 3. Categorical variables presented as count (%).

**eTable 4**. Outcomes for Birth Gestational Age Categories

|  | Premature Gestation | | | | Term Gestation | | | |
| --- | --- | --- | --- | --- | --- | --- | --- | --- |
|  | Placebo (n=93) | Treatment (n=100) | Difference  [95% CI] | Placebo (n=506) | | Treatment (n=498) | Difference  [95% CI] |  |
| 30-day mortality | 1 (1.0%) | 4 (4.0%) | -2.9 [-7.4 to 1.6]% | 5 (1.0%) | | 5 (1.0%) | 0.0 [-1.2 to 1.2]% |  |
| Death or heart transplantation | 6 (6.5%) | 5 (5.0%) | 1.5 [-5.2 to 8.1]% | 18 (3.5%) | | 10 (2.0%) | 1.5 [-0.5 to 3.6]% |  |
| Mechanical Circulatory Support | 3 (3.2%) | 6 (6.0%) | 2.8 [-8.8 to 7.7]% | 22 (4.3%) | | 25 (5.0%) | -0.7 [-3.3 to 1.9]% |  |
| Catheterization or surgical reintervention | 6 (6.5%) | 9 (9.0%) | -2.5 [-10.1 to 5.1]% | 51 (10.0%) | | 38 (7.6%) | 2.4 [-1.1 to 5.9]% |  |
| Bleeding reoperation | 1 (1.1%) | 1 (1.0%) | 0.1 [-2.8 to 3.0]% | 20 (3.9%) | | 6 (1.2%) | 2.7 [0.8 to 4.7]% |  |
| Ranked end point ≥ 91 | 17 (18.3%) | 23 (23.0%) | -4.7 [-16.2 to 6.8]% | 105 (20.6%) | | 80 (16.0%) | 4.6 [-0.1 to 9.4]% |  |

eTable 4. Categorical variables presented as count (%).

**eTable 5**. Outcomes for Chromosomal or Syndromic Diagnosis

|  | No Chromosomal or Syndromic Diagnosis | | | Chromosomal or Syndromic Diagnosis | | |
| --- | --- | --- | --- | --- | --- | --- |
|  | Placebo (n=417) | Treatment (n=399) | Difference  [95% CI] | Placebo (n=183) | Treatment (n=200) | Difference  [95% CI] |
| 30-day mortality | 3 (0.7%) | 4 (1.0%) | -0.3 [-1.6 to 1.0]% | 3 (1.6%) | 5 (2.5%) | -0.9 [-3.7 to 2.0]% |
| Death or heart transplantation | 14 (3.3%) | 10 (2.5%) | 0.8 [ -1.5 to 3.2]% | 10 (5.5%) | 5 (2.5%) | 3.0 [-1.0 to 6.7]% |
| Mechanical Circulatory Support | 16 (3.8%) | 18 (4.5%) | -0.7 [-3.4 to 2.1]% | 9 (4.9%) | 13 (6.5%) | -1.6 [-6.2 to 3.1]% |
| Catheterization or surgical reintervention | 37 (8.9%) | 32 (8.0%) | 0.8 [-3.0 to 4.7]% | 20 (11.0%) | 15 (7.5%) | 3.4 [-2.3 to 9.2]% |
| Bleeding reoperation | 17 (4.0%) | 4 (1.0%) | 3.1 [1.0 to 5.2]% | 4 (2.2%) | 3 (1.5%) | 0.7 [-2.0 to 3.4]% |
| Ranked end point ≥ 91 | 83 (20.0%) | 58 (14.5%) | 5.3 [0.1 to 11]% | 39 (21.3%) | 45 (22.5%) | -1.1 [-9.5 to 7.2]% |

eTable 5. Categorical variables presented as count (%).

**eTable 6.** Postoperative Hospital Length of Stay

|  | **STAT 1-3** | | **STAT 4-5** | | **Neonate** | | **Non-neonate** | | **Premature** | | **Term** | | **- CSD** | | **+CSD** | | |
| --- | --- | --- | --- | --- | --- | --- | --- | --- | --- | --- | --- | --- | --- | --- | --- | --- | --- |
| **PLOS** | P | T | P | T | P | T | P | T | P | T | P | T | P | T | P | T |  |
| </= 5 days | 126 | 141 | 1 | 3 | 3 | 1 | 124 | 143 | 19 | 23 | 107 | 120 | 90 | 105 | 37 | 39 |  |
| 6-10 days | 158 | 164 | 15 | 11 | 27 | 29 | 146 | 146 | 32 | 33 | 140 | 142 | 118 | 117 | 55 | 58 |  |
| 11-15 days | 77 | 80 | 16 | 11 | 32 | 44 | 61 | 47 | 12 | 13 | 81 | 78 | 60 | 62 | 32 | 29 |  |
| 16-20 days | 26 | 28 | 14 | 18 | 23 | 24 | 17 | 22 | 4 | 8 | 36 | 38 | 34 | 28 | 6 | 18 |  |
| 21-25 days | 23 | 21 | 5 | 16 | 15 | 20 | 13 | 17 | 4 | 3 | 24 | 34 | 24 | 24 | 4 | 13 |  |
| 25-30 days | 17 | 13 | 14 | 6 | 18 | 9 | 13 | 10 | 6 | 3 | 25 | 16 | 20 | 11 | 11 | 8 |  |
| 31-35 days | 15 | 4 | 11 | 6 | 19 | 8 | 7 | 2 | 0 | 1 | 26 | 9 | 19 | 8 | 7 | 2 |  |
| 35-40 days | 5 | 11 | 7 | 4 | 10 | 5 | 2 | 10 | 2 | 4 | 10 | 11 | 9 | 5 | 3 | 10 |  |
| 41-45 days | 4 | 4 | 6 | 4 | 7 | 4 | 4 | 4 | 1 | 3 | 10 | 5 | 8 | 7 | 3 | 1 |  |
| 46-50 days | 5 | 4 | 1 | 2 | 3 | 3 | 3 | 3 | 1 | 3 | 5 | 3 | 3 | 4 | 3 | 2 |  |
| 51-55 days | 3 | 2 | 5 | 4 | 6 | 6 | 2 | 0 | 4 | 0 | 4 | 6 | 5 | 5 | 3 | 1 |  |
| 55-60 days | 0 | 4 | 2 | 2 | 1 | 1 | 1 | 5 | 1 | 2 | 1 | 4 | 2 | 2 | 0 | 4 |  |
| 61-65 days | 3 | 3 | 2 | 1 | 1 | 1 | 4 | 3 | 2 | 1 | 3 | 3 | 2 | 1 | 3 | 3 |  |
| 66-70 days | 1 | 4 | 1 | 2 | 1 | 3 | 1 | 3 | 0 | 1 | 2 | 5 | 1 | 1 | 1 | 5 |  |
| 71-80 days | 2 | 0 | 1 | 2 | 1 | 2 | 2 | 0 | 0 | 0 | 3 | 2 | 3 | 1 | 0 | 1 |  |
| 81-90 days | 1 | 2 | 4 | 4 | 3 | 5 | 2 | 1 | 0 | 0 | 5 | 6 | 3 | 6 | 2 | 0 |  |
| 91-100 days | 2 | 0 | 2 | 0 | 1 | 0 | 3 | 0 | 0 | 0 | 4 | 0 | 1 | 0 | 3 | 0 |  |
| >100 days | 10 | 6 | 14 | 11 | 16 | 12 | 9 | 6 | 5 | 2 | 20 | 16 | 15 | 12 | 10 | 6 |  |

**eTable 6**: Categorized postoperative hospital length of stay (PLOS) for each subgroup by those receiving placebo (P) and treatment (T). Each cell contains the number of individuals within each LOS interval bin. *Chromosomal or syndromic diagnosis (CSD).*

**eTable 7**: Outcomes for Patients with Down Syndrome and DiGeorge Syndrome

|  | Down Syndrome | | | DiGeorge Syndrome | | |
| --- | --- | --- | --- | --- | --- | --- |
|  | Placebo (n=94) | Treatment (n=108) | P value | Placebo (n=29) | Treatment (n=18) | P value |
| Death or heart transplantation | 1 (1%) | 3 (3%) | 0.38 | 3 (10%) | 1 (6%) | 0.57 |
| Mechanical circulatory support | 1 (1%) | 5 (5%) | 0.14 | 3 (10%) | 2 (11%) | 0.93 |
| Catheterization or surgical reintervention | 8 (9%) | 4 (4%) | 0.15 | 4 (14%) | 2 (11%) | 0.79 |
| Bleeding reoperation | 1 (1%) | 2 (2%) | 0.64 | 0 (0%) | 0 (0%) | -- |
| PLOS > 30 days | 10 (11%) | 13 (12%) | 0.76 | 11 (38%) | 4 (22%) | 0.26 |


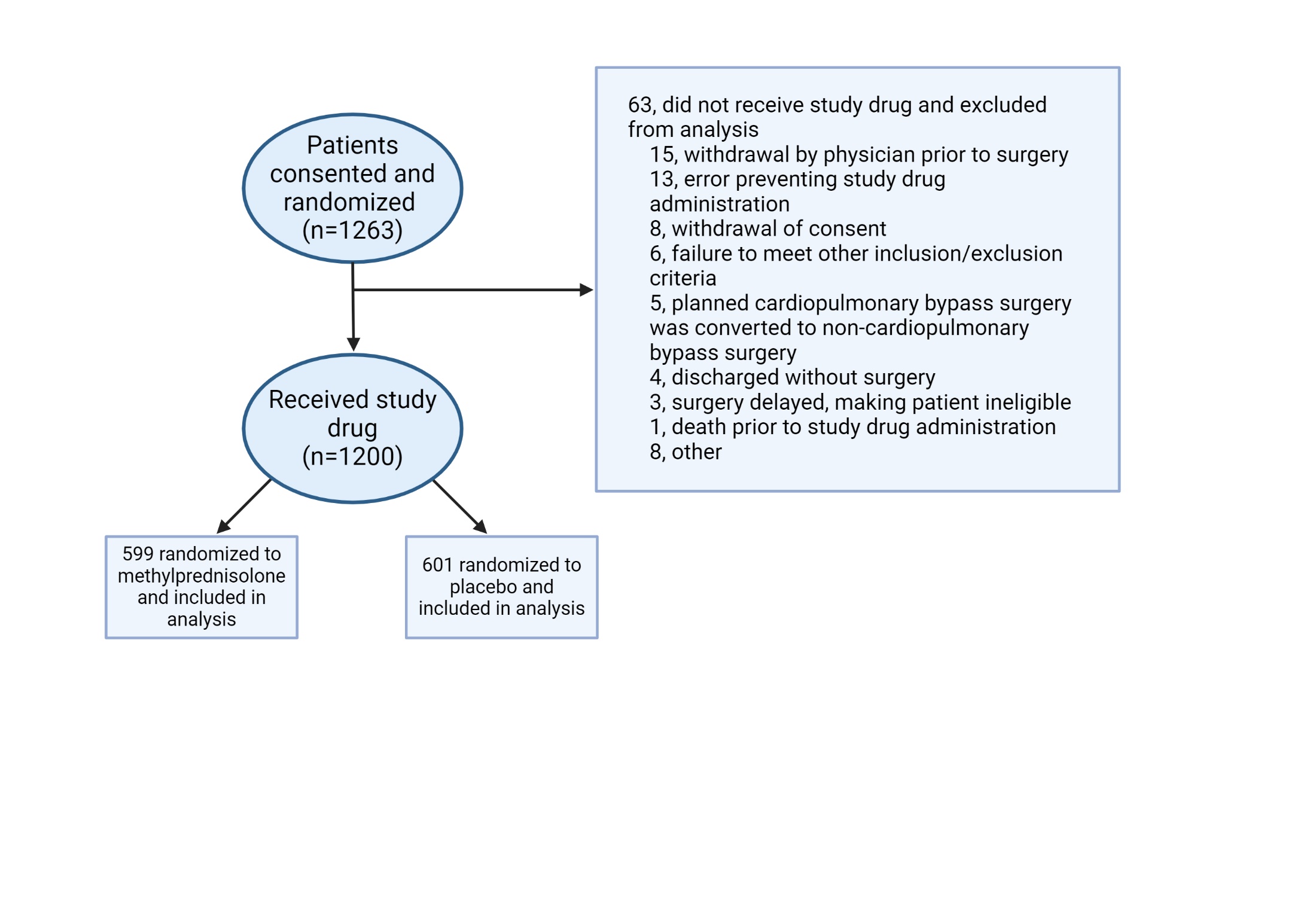
**eFigure 1.** CONSORT Diagram

**eFigure 1.** Patients included within the parent study and reasons for exclusion. Figure created with BioRender.com
